# Opportunities and challenges in incorporating ancillary studies into a cancer prevention randomized clinical trial

**DOI:** 10.1186/s13063-016-1524-9

**Published:** 2016-08-12

**Authors:** Phyllis J. Goodman, Catherine M. Tangen, Amy K. Darke, Kathryn B. Arnold, JoAnn Hartline, Monica Yee, Karen Anderson, Allison Caban-Holt, William G. Christen, Patricia A. Cassano, Peter Lance, Eric A. Klein, John J. Crowley, Lori M. Minasian, Frank L. Meyskens

**Affiliations:** 1 SWOG Statistical Center, Fred Hutchinson Cancer Research Center, 1100 Fairview Ave. N, M3-C102, Seattle, WA 98109 USA; 2 SWOG Statistical Center, Cancer Research and Biostatistics, Seattle, WA USA; 3 Sanders-Brown Center on Aging, University of Kentucky, Lexington, KY USA; 4 Brigham and Women’s Hospital, Harvard Medical School, Boston, MA USA; 5 Division of Nutritional Sciences, Cornell University, Ithaca, NY USA; 6 Arizona Cancer Center, University of Arizona, Phoenix, AZ USA; 7 Glickman Urological and Kidney Institute, Cleveland Clinic, Cleveland, OH USA; 8 Division of Cancer Prevention, National Cancer Institute, Bethesda, MD USA; 9 University of California at Irvine, Orange, CA USA

**Keywords:** Prostate cancer, Ancillary studies, Randomized controlled trial, Study implementation

## Abstract

**Background:**

The Selenium and Vitamin E Cancer Prevention Trial (SELECT) was a randomized, double-blind, placebo-controlled, prostate cancer prevention study funded by the National Cancer Institute and conducted by SWOG (Southwest Oncology Group). A total of 35,533 men were assigned randomly to one of four treatment groups (vitamin E + placebo, selenium + placebo, vitamin E + selenium, placebo + placebo). At the time of the trial’s development, NIH had invested substantial resources in evaluating the potential benefits of these antioxidants. To capitalize on the knowledge gained from following a large cohort of healthy, aging males on the effects of selenium and/or vitamin E, ancillary studies with other disease endpoints were solicited.

**Methods:**

Four ancillary studies were added. Each drew from the same population but had independent objectives and an endpoint other than prostate cancer. These studies fell into two categories: those prospectively enrolling and following participants (studies of Alzheimer’s disease and respiratory function) and those requiring a retrospective medical record review after a reported event (cataracts/age-related macular degeneration and colorectal screening). An examination of the challenges and opportunities of adding ancillary studies is provided. The impact of the ancillary studies on adherence to SELECT was evaluated using a Cox proportional hazards model.

**Results:**

While the addition of ancillary studies appears to have improved participant adherence to the primary trial, this did not come without added complexity. Activation of the ancillary studies happened after the SELECT randomizations had begun resulting in accrual problems to some of the studies. Study site participation in the ancillary trials varied greatly and depended on the interest of the study site principal investigator. Procedures for each were integrated into the primary trial and all monitoring was done by the SELECT Data and Safety Monitoring Committee. The impact of the early closure of the primary trial was different for each of the ancillary trials.

**Conclusions:**

The ancillary studies allowed study sites to broaden the research opportunities for their participants. Their implementation was efficient because of the established infrastructure of the primary trial. Implementation of these ancillary trials took substantial planning and coordination but enriched the overall primary trial.

**Trial registration:**

NCT00006392-S0000: Selenium and Vitamin E in Preventing Prostate Cancer (SELECT) (4 October 2000).

NCT00780689-S0000A:  Prevention of Alzheimer’s Disease by Vitamin E and Selenium (PREADVISE) (25 June 2002).

NCT00784225-S0000B: Vitamin E and/or Selenium in Preventing Cataract and Age-Related Macular Degeneration in Men on SELECT SWOG-S0000 (SEE) (31 October 2008).

NCT00706121-S0000D: Effect of Vitamin E and/or Selenium on Colorectal Polyps in Men Enrolled on SELECT Trial SWOG-S0000 (ACP) (26 June 2008).

NCT00063453-S0000C: Vitamin E and/or Selenium in Preventing Loss of Lung Function in Older Men Enrolled on SELECT Clinical Trial SWOG-S0000 (26 June 2003).

**Electronic supplementary material:**

The online version of this article (doi:10.1186/s13063-016-1524-9) contains supplementary material, which is available to authorized users.

## Background

The Selenium and Vitamin E Cancer Prevention Trial (SELECT) was a randomized, double-blind, placebo-controlled, prostate cancer prevention study funded by the National Cancer Institute (NCI) and the National Center for Complementary and Alternative Medicine (NCCAM) and conducted by SWOG (formerly known as the Southwest Oncology Group) [1]. Between August 2001 and June 2004, 35,533 men were assigned randomly to one of four groups: vitamin E + placebo, selenium + placebo, vitamin E + selenium, placebo + placebo. Accrual to the trial came from more than 400 study sites in the US, Puerto Rico, and Canada. On 15 September 2008, the independent Data and Safety Monitoring Committee (DSMC) recommended discontinuation of study supplements because of lack of efficacy for risk reduction, and because futility analyses demonstrated no possibility of benefit from the supplements to the anticipated degree, i.e., 25 % reduction in prostate cancer incidence, with continued supplementation and additional follow-up [2]. A CONSORT diagram for the primary analysis is presented in Additional file 1. An updated analysis [3] in October 2011 revealed that the men who had been taking vitamin E and the selenium placebo had a 17 % increased risk of prostate cancer compared to the men who took two placebos (*p* = .008). The study sites ended their participation, and 17,781 participants agreed to continue providing regular health updates to the Statistical Center in the Centralized Follow-up (CFU) [4] phase.

At the time of the development of SELECT, the National Institutes of Health (NIH) had invested substantial resources in evaluating the use of nutritional supplements. In particular, there was scientific interest in the potential benefits of antioxidants, including funding for large trials such as the Heart Outcomes Prevention Evaluation (HOPE), which examined the effects of vitamin E on cardiovascular risk in a high-risk population, and the Age-Related Eye Disease Study (AREDS), which examined the effects of vitamin C, vitamin E, beta-carotene, and zinc on the progression of age-related macular degeneration. Given the size of SELECT, there was a desire to capitalize on the knowledge gained from following a large cohort of healthy, aging males on the effect of selenium and/or vitamin E on other disease endpoints within the trial.

From the perspective of the funding agencies, ancillary studies are a cost-effective mechanism to answer important questions in the same population as the randomized trial, using pre-existing clinical study sites with reduced recruitment efforts and use of existing study data. The purpose of this paper is to describe both the opportunities and challenges of incorporating ancillary studies into an active clinical trial.

## Methods

SELECT was coordinated by SWOG, a national consortium of institutions and investigators that conducts multidisciplinary clinical trials to improve the practice of medicine in preventing, detecting, and treating cancer, and to enhance the quality of life for cancer survivors. Primary support for SWOG comes from the National Cancer Institute (NCI). Institutions across the US that enroll patients into SWOG group studies include those affiliated with major academic medical centers and their associated affiliate institutions, and Community Clinical Oncology Program (CCOP) sites, which are community hospitals or consortia with a mandate for both clinical research and cancer control, including prevention research.

Recruitment to SELECT was from SWOG, other cooperative oncology groups, the Department of Veterans Affairs (VA), study sites that had successfully participated in other large prevention trials, as well as 13 Canadian institutions. Within the cooperative groups, CCOPs provided 30 % of the total accrual.

Researchers with an interest in launching an ancillary study were asked to submit a proposal to the SELECT Executive Committee, comprising SWOG, SELECT, and NCI leadership, for review. Proposals were reviewed for scientific merit and feasibility, and were assessed in terms of required sample size, participant characteristics of the target population, data collection methodology and potential impact on the primary trial. Funding was required to cover costs of the study sites to do the work required of the ancillary study, as well as the costs of the SELECT Statistical Center to implement the required changes to the existing study and general study support.

Four ancillary trials were activated within SELECT: (1) Prevention of Alzheimer’s Disease with Vitamin E and Selenium (PREADVISE), (2) the SELECT Eye Endpoint (SEE) study, (3) the Respiratory Ancillary Study (RAS), and (4) the Adenomatous Colorectal Polyps (ACP) study. Common characteristics of these studies included study objectives independent of prostate cancer prevention with hypotheses that involved either selenium, vitamin E, or the combination of the two. Each ancillary study had a project team that was independent from SELECT and was responsible for protocol development and a chapter for the SELECT Study Manual. They used the SELECT participant data, specimens and data management, and were comanaged by SELECT and the independent ancillary study teams. Each ancillary study required Institutional Review Board (IRB) approval by participating study sites and written informed consent from participants. The SELECT Statistical Center worked with the ancillary study teams on issues related to data transfers and reports to facilitate study and participant management.

Potential opportunities that could result from the addition of the ancillary studies included (1) increased research options for participants, (2) enhanced accrual to the primary trial, and (3) improved adherence to the primary trial. In order to evaluate the impact of adherence to SELECT we implemented a Cox proportional hazards model, where time until the participant went off treatment was the dependent variable and participation in an ancillary study was a time-dependent covariate because enrollment could have been post randomization to SELECT. This analysis was limited to the two ancillary studies that required active follow-up (PREADVISE and RAS). Participants who went off treatment for reasons other than the development of prostate cancer or death and prior to the release of study results and suspension of study supplements (23 October 2008) were considered an event; participants who went off treatment due to either development of prostate cancer, death or the study termination were censored at the time of their competing event. Covariates included in the model were age (50–64 years versus 65–74 years versus 75 years and older), African American race (yes/no), Hispanic ethnicity (yes/no), whether the participant was enrolled at a site that participated in either PREADVISE or RAS (yes/no), smoking status (current versus never/former), education (college or greater versus other), marital status (married versus other), and BMI (<25 versus 25–29.9 versus ≥30). Interaction terms for ancillary study participation, race, ethnicity, and age were also included.

All men randomized on SELECT who provided informed consent were included in the analysis excluding men who (1) had a pre-randomization diagnosis of prostate cancer or (2) were from sites in Puerto Rico. Puerto Rican sites were excluded due to the small sample size. Analysis was done in SAS version 9.2 using PROC PHREG.

In addition to the opportunities, there was also the anticipation of individual study challenges as well as some that would bridge all of the trials. These included (1) initial activation of the trial, (2) buy-in from the study sites, (3) integration of study procedures, (4) communication, and (5) study monitoring. The early closure of SELECT was another unanticipated challenge.

## Results

The four ancillary studies implemented within SELECT fell into one of two categories: (1) a study requiring active interaction with the participant with prospective data collection or (2) a study requiring a retrospective medical record review based on a reported non-prostate cancer event (Table 1).

**Table 1:** SELECT and the approved ancillary studies

	Primary endpoint	Activation date	Last registration date	# men	# sites	Study site recruitment base	Follow-up procedure
SELECT	Prostate cancer	July 2001	June 2004	35,533	427	Cooperative Group; Veterans Admin Cooperative Studies program; sites involved in other large prevention trials (WHI, HOPE); Canadian urologic sites	Active follow-up every 6 months
PREADVISE	Alzheimer’s disease	May 2002	Sept 2009	7553	128	All invited	Active follow-up/memory screens
RAS	COPD	June 2004	April 2007	2921	18	20 sites with high proportion of smokers	Active follow-up/pulmonary function tests
SEE	Cataracts and AMD	July 2004	Dec 2009	2436	105	All invited	Medical record review
ACP	Colorectal adenomas	June 2008	May 2013	8089	76	100 sites with large number of men; men on Centralized Follow-up	Medical record review

*ACP* Adenomatous Colorectal Polyps study, *AMD* age-related macular degeneration, *COPD* chronic obstructive pulmonary disease, *PREADVISE* Prevention of Alzheimer’s Disease with Vitamin E and Selenium, *RAS* Respiratory Ancillary Study, *SEE* SELECT Eye Endpoint study

### Studies requiring active participation

#### PREADVISE

The first ancillary study activated was the Prevention of Alzheimer’s Disease by Vitamin E and Selenium (PREADVISE), funded by the National Institute on Aging [5–7]. The objective of this study was to evaluate the effect of selenium and vitamin E in combination and alone on the reduction of the clinical incidence of Alzheimer’s disease (AD). Registered participants were screened annually for memory issues with the Memory Impairment Screen (MIS) [8]. For those men who failed the MIS screen, a second stage of screening consisting of an expanded mental status examination using a subset of the Consortium to Establish a Registry for Alzheimer’s Disease (CERAD) battery of mental status tests [9] was administered by study site staff and results were sent to the PREADVISE Coordinating Center. Failure on both the brief and expanded screenings suggests problems with cognition and memory. These participants were asked to sign a second consent form to obtain a medical work-up for treatable causes of memory loss. The age eligibility criteria for PREADVISE was older than that for SELECT (62 versus 55 years) which was later lowered to 60 years or above for African Americans. The accrual goal was 10,400 participants from all SELECT study sites.

#### RAS

The Respiratory Ancillary Study (RAS) was funded by the National Heart, Lung and Blood Institute and also required active participation [10, 11]. The objective of this study was to understand whether vitamin E and/or selenium can slow the loss of lung function that occurs naturally with aging. The accrual goal was 3000 participants, with as many current smokers as possible. Participants were followed with regular pulmonary function tests (PFT).

### Studies requiring medical record review

#### SEE

The SELECT Eye Endpoints (SEE) study was funded by the National Eye Institute [12]. The accrual goal was for 700 men who had age-related macular degeneration (AMD) events and 2240 men with cataract events. Men at participating study sites who reported the development of a cataract post randomization, or a diagnosis of AMD by responding positively to vision-related questions, were approached to participate in this study. Consenting participants provided a Medical Release Form, and the SEE Statistical Center gathered the records for review.

#### ACP

The Adenomatous Colorectal Polyps (ACP) study was funded by the NCI [13]. Men who reported a colorectal endoscopic screening procedure (colonoscopy or sigmoidoscopy) were asked to participate. The primary aim was to determine whether the study interventions reduced colorectal adenoma occurrence. Adenoma recurrence was determined by review of endoscopy records followed by confirmation from histology reports of the presence of one or more adenomas. The study site procedures were similar to those for SEE, with the addition of a required tissue sample. The goal was to capture 8000 procedures.

### Opportunities

#### Increased study options for participants

The ability of participants to register to the ancillary studies depended on the interest of their local study site. The level of study site participation in the ancillary studies varied greatly and is discussed in detail later. For two of the studies, RAS and ACP, the sites with the largest accrual to SELECT were specifically recruited to participate as they provided the biggest potential return for the time and money invested in starting up a new study. The smaller sites had the opportunity to participate in two studies (PREADVISE and SEE) but as can be seen in Table 2 only 22 % of the smallest sites (1–25 participants) chose to participate. For medium size sites (26–100 participants), 51 % of the sites participated in at least one ancillary study.

**Table 2:** Number of ancillary studies in which study sites participated by size of study site

		Number of ancillary studies				
Number of participants on SELECT	*N* sites of this size	None *n* (%)	One *n* (%)	Two *n* (%)	Three *n* (%)	Four *n* (%)
≤25 participants	167	130 (78 %)	23 (14 %)	6 (4 %)	8 (5 %)	0 (0 %)
26–100 participants	172	84 (49 %)	48 (28 %)	30 (17 %)	10 (6 %)	0 (0 %)
101–500 participants	74	20 (27 %)	22 (30 %)	18 (24 %)	13 (18 %)	1 (1 %)
>500 participants	12	2 (17 %)	2 (17 %)	3 (25 %)	2 (17 %)	3 (25 %)
Total	425	236	95	57	33	4

**Fig. 1:**
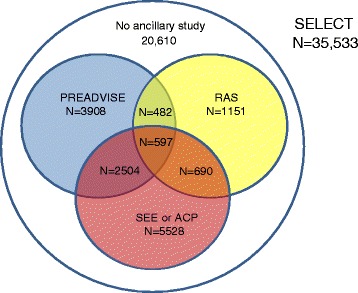
Overlap of participation in the ancillary studies

Registrations to the ancillary studies took place throughout the course of SELECT (Table 3). Ultimately 14,923 men, 42 % of the SELECT population, participated in at least one of the ancillary studies; 597 men participated in all four. The overlap of the participation in the studies can be seen in Fig. 1. For ease of presentation, the two studies only requiring medical record review are grouped together.

**Table 3:** Timing of registration to ancillary study and randomization to SELECT

	Years between randomization and registration to ancillary study (number of men)^a^								
	0^b^	1	2	3	4	5	6	7	Post SELECT unblinding
PREADVISE	2095	2216	952	185	428	815	416	110	267
SEE	0	68	182	355	535	424	290	67	510
RAS	141	489	1125	1157	7	1	0	0	0
ACP	0	0	0	0	15	75	100	96	7642

^a^Prior to study unblinding and cessation of supplementation

^b^Within 45 days of randomization

*ACP* Adenomatous Colorectal Polyps study, *PREADVISE* Prevention of Alzheimer’s Disease with Vitamin E and Selenium, *RAS* Respiratory Ancillary Study, *SEE* SELECT Eye Endpoint study

#### Improved adherence to primary trial

Participant characteristics for all of SELECT and for the two ancillary studies requiring active participation are presented in Table 4. The biggest differences are in the age distribution, race, and smoking status due to differing registration requirements. After accounting for the covariates, participation in an ancillary study improved adherence to the primary trial, as defined by time until the participant went off both study supplements. The hazard ratio for time until off for those participating at a site which participated in either PREADVISE or RAS was 0.80 (95 % confidence interval 0.75–0.86, *p* < .0001), meaning that the rate of going off study supplements was 20 % less for participants whose site was involved in ancillary studies than for those that were not at such sites.

**Table 4:** Participant characteristics

	SELECT		PREADVISE		RAS	
Age (years)						
Median (IQ range)	62	(58,67)	65	(61,69)	61	(57,66)
50–54	1480	4.2 %	38	0.5 %	207	7.1 %
55–64	20,347	58.3 %	3567	47.7 %	1784	68.2 %
65–74	10,808	31.0 %	3260	43.6 %	795	27.2 %
75+	2252	6.5 %	619	8.3 %	134	4.6 %
Race/ethnicity						
White	27,571	79.0 %	6121	81.8 %	2006	68.7 %
African American	4286	12.3 %	660	8.8 %	694	23.8 %
Hispanic (non-African American)	1196	3.4 %	198	2.7 %	73	2.5 %
Hispanic (AA)	356	1.0 %	31	0.4 %	19	0.7 %
Other	1478	4.2 %	474	6.3 %	128	4.4 %
Education						
≤ High school graduate	7682	22.0 %	1560	20.8 %	674	23.1 %
Some college/vocational school	9355	26.8 %	1960	26.2 %	850	29.1 %
≥ College graduate	17,515	50.2 %	3931	52.5 %	1371	47.0 %
Unknown	335	1.0 %	33	0.4 %	25	0.9 %
Marital status						
Currently married	28,430	81.5 %	6365	85.1 %	2189	75.0 %
Not married	6272	18.0 %	1097	14.7 %	711	24.4 %
Unknown	185	0.5 %	22	0.3 %	20	0.7 %
Smoking status						
Current	2682	7.7 %	398	5.3 %	470	16.1 %
Former	17,156	49.2 %	3831	51.2 %	1393	47.7 %
Never	14,882	42.7 %	3244	43.4 %	1041	35.7 %
Unknown	167	0.5 %	11	0.2 %	16	0.6 %
BMI						
< 25	6967	20.0 %	1504	20.1 %	598	20.5 %
25–30	16,751	48.0 %	3662	48.9 %	1357	46.5 %
≥ 30	10,970	31.4 %	2292	30.6 %	957	32.8 %
Unknown	199	0.6 %	26	0.4 %	8	0.3 %

*BMI* body mass index, *IQ* interquartile

### Challenges

#### Study activation

Accrual to SELECT took two fewer years than originally planned. Because of the start-up and planning time needed for the ancillary studies, the rapid accrual to SELECT meant that activation of the ancillary trials happened after SELECT randomizations began. This was particularly difficult for the trials that actively followed participants and their need for baseline, pre-randomization data.

The first registration to PREADVISE was 10 months after SELECT began randomizing participants. An extra 2 years of accrual would have given the staff at the study sites additional time to learn and incorporate study procedures that were different than those for an oncology trial. As it became apparent that the SELECT accrual period would be shorter than planned, it became clear that the accrual goal for PREADVISE would not be reached. At the time SELECT closed accrual, 3858 men had been registered to PREADVISE. Changes were made to the PREADVISE eligibility requirements that allowed registration to PREADVISE after the participant was randomized to SELECT. The sample size was also modified downwards to 6500.

RAS was activated the month that SELECT accrual ended. Because the participants were to be accrued after randomization to SELECT, and no baseline measures would be available, the RAS incorporated objectives that were assessed in both longitudinal and cross-sectional analyses while taking advantage of the randomization. Participants were followed annually for 3 years; these visits coincided with the SELECT annual visits.

For the two studies that involved submission of source documentation after a reported event during SELECT follow-up, the rapid accrual was not an issue.

#### Buy-in from study sites and participant accrual

As the first activated ancillary study, PREADVISE was the first to experience the reluctance of some of the study sites to participate. Sites were surveyed as to their interest in participating and to identify barriers to participation. The primary barriers were lack of staff time, perceived complexity of baseline and follow-up requirements, burden on SELECT study site staff, lack of familiarity with memory impairment screening and cognitive endpoints, lack of interest by the site principal investigator (PI), and inadequate compensation. Regulatory and IRB issues were related to absence of language regarding the potential risks, consent for the use of SELECT specimens for the genetic screening of *ApoE* alleles, and the re-consent process for participants who met the criteria for cognitive impairment. Efforts to make PREADVISE appeal to more study sites included reducing data requirements, in-person training opportunities, the addition of receptions at the SELECT semiannual training workshops for participating clinical research associates (CRAs), the addition of tools to assist with the registration and follow-up processes and increased site payments which included both a per capita reimbursement as well as funds to support the process of getting the protocol passed by local IRBs. Ultimately, 152 sites said they would participate in PREADVISE but only 128 of 427 (30 %) of sites registered participants. The final accrual was 7553.

All sites were encouraged to participate in SEE and it was expected that most sites would participate due to the limited additional work to register participants. However, the anticipated time and effort needed to accomplish additional tasks, such as obtaining local IRB approval, explaining the study to potential participants, administering a new informed consent and assisting the participant in completing the Medical Release Form, was considered by many sites to be insufficiently covered by the reimbursement. Sites were surveyed as to whether or not a modest increase in the amount of support would change their ability to participate. However, survey results indicated that the proposed payment increase would not have a substantial impact on study site participation. The large majority of sites that participated in SEE felt that the methodology was simple and easy to implement and did not place an excessive burden on staff. Ultimately, 148 study sites obtained IRB approval with 105 of these study sites registering 2436 men to the study. Among the registered men, there were enough cataract endpoints but the goal for the AMD endpoint was not reached.

The requirements to be a RAS site were identified prior to trial activation. The smaller sample size, the need for smokers and the need for training on administering the pulmonary function tests (PFT) resulted in a limited number of study sites that were invited to join RAS. Site selection was upfront and based on the number of registered participants on SELECT, the number of smokers, and the interest of the study site PI. A total of 18 sites participated, registering 2920 men with 16.1 % being current smokers compared to 7.5 % on SELECT.

ACP learned from SEE that it would be more efficient to focus recruitment efforts on a smaller number of sites. The top accruing sites to SELECT were invited to participate, resulting in IRB approval of 90 study sites covering 16,899 participants. By the time the SELECT study sites had closed, 4794 men had been registered, short of the accrual goal of 8000.

#### Integration of study procedures and training

A key component to ensuring successful implementation of the ancillary studies was their full integration into SELECT procedures. This included using the SWOG format for the protocol and Informed Consent Form as well as using the central participant registration program and study forms that complied with SELECT guidelines. Each ancillary study team was responsible for a chapter in the SELECT Study Manual outlining study procedures at the study sites, including participant recruitment and follow-up, data management, endpoint reporting, and any additional biologic specimen needs. In order to integrate the ancillary studies as seamlessly as possible, all forms were developed using the same software, and were maintained along with the ancillary Study Manual on the SELECT website.

For PREADVISE and RAS, another integration issue was how and when to present the ancillary study to potential participants without overburdening the participant with too much information. A participant was introduced to PREADVISE at the discretion of the CRA at his study site either at the participant’s SELECT randomization visit or at any subsequent visit. This flexibility also allowed time for younger participants to become age-eligible for PREADVISE. Because of study activation issues, RAS was also introduced to participants at a post-baseline visit which also reduced the amount of information presented to the participants at any one time.

Training opportunities provided by the ancillary studies included those directed at study site staff and others aimed directly at the participants. In addition to their sections in the SELECT Study Manual which contained training materials, each ancillary study had sessions at the SELECT semi-annual training workshop which included large didactic presentations, smaller break-out sessions, open forums, poster sessions, and informal gatherings where the CRAs had a chance to interact with ancillary study staff. Other training tools were by the ancillary study staff and were dependent on available funding and staff time for development (Table 5). Coordinating this effort across the primary trial and the four ancillary trials was challenging but resulted in dynamic training workshops.

**Table 5:** Training, adherence, and retention tools and their use by each ancillary study

	PREADVISE	RAS	SEE	ACP
Study Manual	X	X	X	X
Workshop	X	X	X	X
In-person study site training		X		
Study website for participants	X			
Participant newsletter	X			
Staff newsletter/tip sheets/updates		X		X
Participant incentive items	X	X		
Staff incentive items	X	X		
Conference calls with study sites	X	X		

*ACP* Adenomatous Colorectal Polyps study, *PREADVISE* Prevention of Alzheimer’s Disease with Vitamin E and Selenium, *RAS* Respiratory Ancillary Study, *SEE* SELECT Eye Endpoint study

#### Communication

As with any large collaborative effort, communication between all interested parties is paramount to success but can be difficult to achieve. A variety of tools were in place to facilitate communication with the study sites and between the SELECT team and the ancillary study teams. The SELECT Workbench, the on-line collection of protocols, manual, documents, and procedures used for all aspects of the management of SELECT, was maintained by the SELECT Statistical Center and was the portal for participant registrations and data submission for the ancillary studies. As a result, there was a need for constant communication between the ancillary study teams and the SELECT Statistical Center concerning ancillary study data collection, materials provided on the website, and study status. Additionally, various reports were developed and produced routinely for the coordinating centers of the ancillary studies including (1) study site IRB approval status, (2) lists of potentially eligible and registered participants, (3) monthly accrual and basic demographics, and (4) data needed for evaluation of the study. These reports were generally run in batch and placed on secure File Transfer Protocol (FTP) sites. Evaluated endpoint data collected from the external ancillary study teams were put on the secure FTP sites, uploaded to the secure SWOG database, and were used by the SELECT statistical staff to prepare the annual DSMC reports.

Conference calls with the SELECT study team and the ancillary study teams were held on a regular basis (PREADVISE and ACP) as well as on an as-needed basis (SEE and ACP). The calls were an opportunity to address implementation issues and other time-sensitive concerns. They also more generally served the purpose of providing an avenue for open conversation between the groups.

Ancillary study PIs were incorporated into the study leadership by serving as members of the SELECT Steering Committee and joining the Executive Committee monthly calls as needed. They also had periodic meetings with NCI leadership to speak about unique study issues without the immediate SELECT leadership present.

Communication with the study site staff regarding data collection on the ancillary studies was done through the SELECT Statistical Center, as it housed the data; for other procedural issues and ancillary-specific training issues the study site staff were directed to the ancillary study team.

#### Monitoring

Members with expertise in the disease area of the ancillary studies were added to the SELECT DSMC so that it could function as the DSMC for the ancillary studies. Data from the ancillaries and SELECT were monitored at their annual meeting. There were no explicit predetermined guidelines regarding what would happen to the ancillary studies in case of early closure of SELECT. The ancillary study PIs were concerned that endpoints for their studies might not be completed and the investments in their studies would not be fulfilled should the primary trial end with extreme positive or negative results. Ultimately, the trial intervention ended early, and the decision to continue participant follow-up via the CFU was made in part to allow time for the ancillary studies to gather as much additional data as possible to answer their study questions.

#### Impact of early closure of SELECT and transition to Centralized Follow-up

After the early release of study results, SELECT study sites continued to follow their participants with in-person visits. Follow-up transitioned to a CFU model [4] and 17,607 men agreed to be followed annually. Due to the status of each of the ancillary studies, the shift in how participants were followed impacted each differently.

PREADVISE had not finished collecting their primary study data and needed continued follow-up. Procedures were modified so that staff at their Coordinating Center could collect follow-up by telephone using the Telephone Interview for Cognitive Status-Modified (TICS-M) [14] to replace the MIS and a Long Memory and Thinking Screen (LMTS). This entailed a major protocol amendment with a new Informed Consent Form and the subsequent secure transfer of personal identifiers (including name, telephone number) from the SELECT Statistical Center to enable them to contact participants directly. Of the 6957 PREADVISE men who were alive and willing to be followed by their study site, 62 % agreed to be contacted by staff at the PREADVISE Coordinating Center.

The transition to CFU created some opportunities for PREADVISE. The ability to communicate directly with the participants allowed PREADVISE to establish its own identity with the men. Updates to participant survival status from these contacts were transmitted back to the SELECT Statistical Center. The transition also afforded PREADVISE the ability to have more control of the study procedures including the ability to utilize trained examiners for administration of the MIS and follow-up procedures for the participants being followed [15, 16].

For RAS, when study supplementation stopped, the PFT endpoint was available for only 57 % of their participants, resulting in the loss of power for the primary analysis of lung function decline over 3 years. The final PFTs were done while the participants were off supplements and still being followed by the study sites.

At the time of the transition to CFU, ACP had accrued about half of its goal. Men who were registered to the SELECT CFU but had been at sites not participating in ACP were now able to register. A letter of invitation, Medical Release and Informed Consent Forms were sent to men who had reported a colorectal screening procedure. This approach yielded an additional 3303 participants for a total of 8097 participants, enabling the trial to meet its accrual goal.

SEE had accrued enough men to answer its cataract objective but did not have enough AMD endpoints. The CFU provided the ability to identify additional incident AMD cases from men not at SEE sites.

## Discussion

The incorporation of ancillary studies into a large ongoing randomized trial presents both opportunities and challenges. The concentration of research resources including the identification of study sites, recruitment plans, training venues, data and study management procedures, and overall administrative and statistical support makes for an efficient study model. These studies also enhance collaboration between researchers interested in similar agents across a variety of disease areas.

For the ancillary studies, the advantages to joining forces with an existing trial were clear. Advantages to the primary trial were also evident, specifically, increased recruitment potential, improved adherence, and the ability of study sites to broaden research opportunities for their participants. SELECT recruited its 35,533 men 2 years ahead of schedule and prior to the full implementation of some of the ancillary studies, such that the impact of ancillary studies on accrual to the main trial was minimal. The impact on adherence to the primary trial was substantial; men who were at sites that participated in an ancillary study were 20 % less likely to stop study supplements. However, this analysis was limited because we may not have adequately captured participant factors that could have influenced registration to the ancillary studies or study site factors that may also have been important.

There were challenges in these collaborations. SELECT was ultimately a prostate cancer prevention trial and study implementation procedures were driven by this endpoint. As a result, for the ancillary studies, there was a loss of control of study and participant management, as it was necessary that their procedures fit within the primary trial. For some ancillaries this entailed an increase in the complexity of procedures that were otherwise simple and straightforward to implement. There were also issues of integrating a non-cancer study into an oncology setting (e.g., reluctance of CRAs to collect different types of data) and the need to conform to NCI and SWOG requirements. Changes in study site leadership also proved challenging when a new study site PI was not interested in or committed to the ancillary study and did not provide adequate support to implement the study.

For the SELECT Statistical Center, the ancillary studies provided additional funding to cover tasks necessary for their successful integration into SELECT. However, the additional tasks to implement and coordinate the studies were greater than had been expected and funding did not fully cover the increased workload.

The early closure of SELECT directly impacted the ancillary studies. In addition, had there been either an extreme positive or negative result of one of the ancillary studies prior to the closure of SELECT, the DSMC would have had to decide what to do with the primary trial. While there were no guidelines for this situation, the DSMC had been monitoring outcome data from the primary trial and the ancillary studies since their inception, and thus had the global picture of all of the trials and would have been in a good position to make this decision. The continued responsibility to publish the trial results and for ongoing data analyses of both the primary trial and the ancillary studies requires persistence by the remaining staff to ensure that the investment of this large collaboration yields as much science as possible.

## Conclusion

As a general population study, SELECT presented opportunities to look at other health areas. These ancillary study endpoints were not secondary outcomes of the primary trial but rather were independent primary analyses in imbedded trials. Ancillary studies as add-ons to clinical trials provide the opportunity to conduct research using an existing infrastructure. Such studies are encouraged by the NIH and at any given time there is a multitude of funding opportunities for such studies through the individual institutes. The benefits to the parent trial can be tangible in terms of recruitment and adherence but also extend beyond the time of the active intervention and follow-up. The benefits and potential drawbacks to the ancillary studies should be weighed and discussed with the primary statistical center prior to joining the main study, for the benefit of all researchers and participants.

## Abbreviations

ACP, Adenomatous Colorectal Polyps; AD, Alzheimer’s disease; AMD, age-related macular degeneration; AREDS, Age-Related Eye Disease Study; CCOP, Community Clinical Oncology Program; CERAD, Consortium to establish a Registry for Alzheimer’s Disease; CFU, Centralized Follow-up; CRA, clinical research associate; DSMC, Data and Safety Monitoring Committee; FTP, File Transfer Protocol; HOPE, Heart Outcomes Prevention Evaluation; IRB, Institutional Review Board; LMTS, Long Memory and Thinking Screen; MIS, Memory Impairment Screen; NCCAM, National Center for Complementary and Alternative Medicine; NCI, National Cancer Institute; NIH, National Institutes of Health; PFT, pulmonary function tests; PI, principal investigator; PREADVISE, Prevention of Alzheimer’s Disease with Vitamin E and Selenium; RAS, Respiratory Ancillary Study; SEE, SELECT Eye Endpoint; SELECT, Selenium and Vitamin E Cancer Prevention Trials; TICS-M, Telephone Interview for Cognitive Status-Modified; VA, Veterans Affairs

## Additional files

Additional file 1: Consort diagram for primary analysis of SELECT. (PDF 9 kb) Additional file 2: Participating IRBs on SELECT. (XLS 81 kb)
