# Supplementary material for: Opportunities and challenges in incorporating ancillary studies into a cancer prevention randomized clinical trial
Source: Trials. 2016 Aug 12;17:400. doi: 10.1186/s13063-016-1524-9 (PMC4983010; doi:10.1186/s13063-016-1524-9)
Supplement: Additional file 1: — Consort diagram for primary analysis of SELECT. (PDF 9 kb) [file 13063_2016_1524_MOESM1_ESM.pdf]

Consort Diagram for primary analysis of SELECT

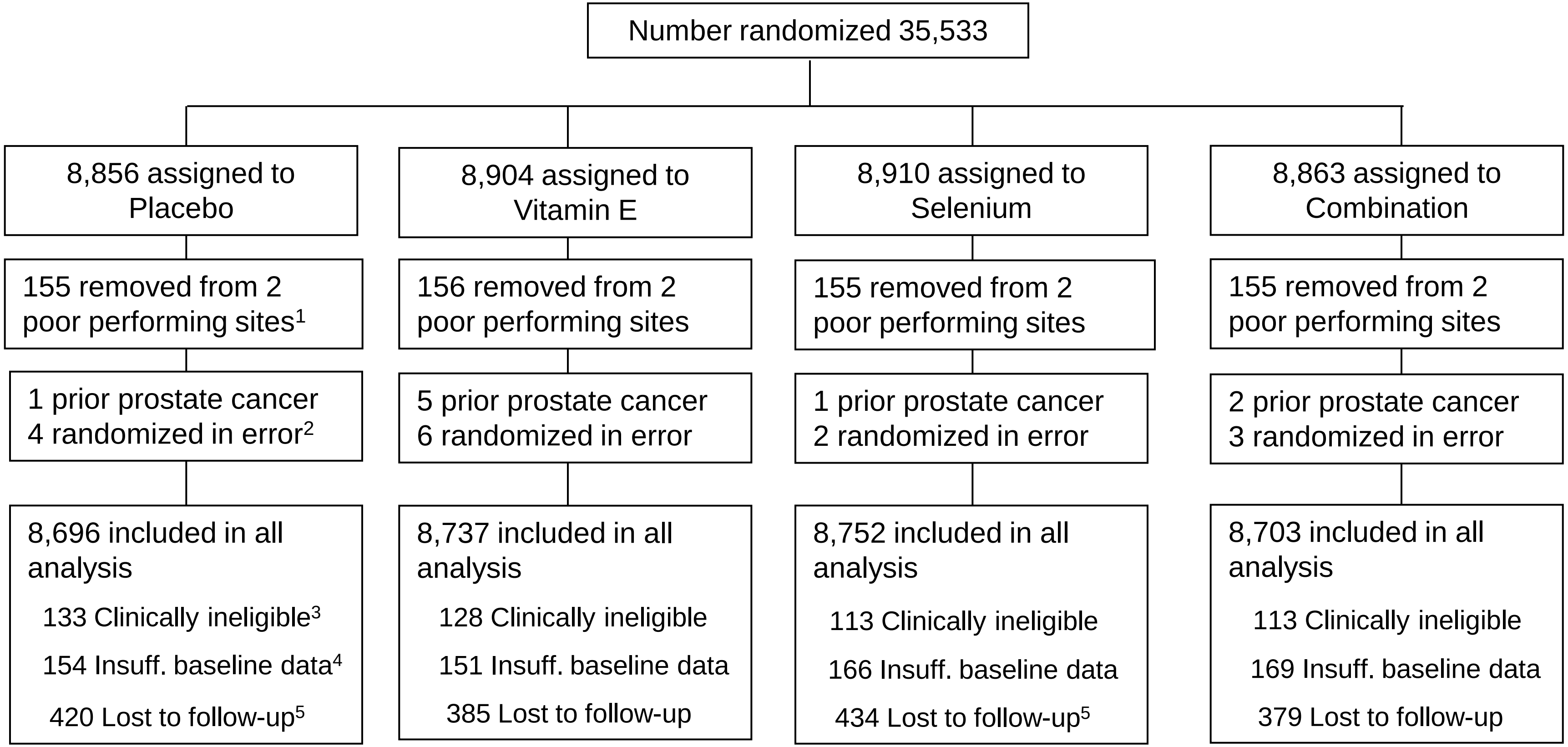

1: All participants from 2 sites removed due to poor data participant management, data management, and regulatory issues.

2: Proper informed consent never received.

3: Clinically ineligible due to elevated blood pressure, high grade PIN, suspicious DRE or elevated PSA, aspirin dosage, prior cancer < 5 years prior to randomization, participation in another clinical trial or other clinical reason. These men were included in all analyses.

4: Insufficient baseline data to completely evaluate clinical eligibility. Blood pressure, PSA and/or DRE not done within required time frame (but normal) or other data-related reason. These men were included in all analyses.

5: Last contact date > 24 months prior to analysis. All data up until their last contact are included in all analyses. For time-to-event analyses, these men are censored at their last follow-up. These men could also have been either clinically ineligible or had insufficient baseline data.
